# A bayesian spatio-temporal dynamic analysis of food security in Africa

**DOI:** 10.1038/s41598-024-65989-z

**Published:** 2024-07-02

**Authors:** Adusei Bofa, Temesgen Zewotir

**Affiliations:** School of Mathematics, Statistics, and Computer Science, University of KwaZulu Natal, Oliver Tambo Building, Westville Campus, Durban, South Africa

**Keywords:** Child care factor, Dynamic parameters, Markov Chain Monte Carlo (MCMC), Food Insecurity Experience Scale, Principal Component Analysis(PCA), Undernourishment, Statistics, Environmental social sciences, Sustainability

## Abstract

Exploring the factors influencing Food Security and Nutrition (FSN) and understanding its dynamics is crucial for planning and management. This understanding plays a pivotal role in supporting Africa's food security efforts to achieve various Sustainable Development Goals (SDGs). Utilizing Principal Component Analysis (PCA) on data from the FAO website, spanning from 2000 to 2019, informative components are derived for dynamic spatio-temporal modeling of Africa’s FSN Given the dynamic and evolving nature of the factors impacting FSN, despite numerous efforts to understand and mitigate food insecurity, existing models often fail to capture this dynamic nature. This study employs a Bayesian dynamic spatio-temporal approach to explore the interconnected dynamics of food security and its components in Africa. The results reveal a consistent pattern of elevated FSN levels, showcasing notable stability in the initial and middle-to-late stages, followed by a significant acceleration in the late stage of the study period. The Democratic Republic of Congo and Ethiopia exhibited particularly noteworthy high levels of FSN dynamicity. In particular, child care factors and undernourishment factors showed significant dynamicity on FSN. This insight suggests establishing regional task forces or forums for coordinated responses to FSN challenges based on dynamicity patterns to prevent or mitigate the impact of potential food security crises.

## Introduction

The spatio-temporal technique has garnered considerable attention in the twenty-first century due to its widespread application across various research fields^1,2^. The methodology employed in modeling spatiotemporal phenomena enables the capture of intricate dynamics and interactions among time, space, and numerous components (factors) within a given system, such as food security and nutrition (FSN)^3^. A focal point in the global public health domain revolves around the management and assurance of food security and nutrition^4^. Policymakers and researchers are actively exploring mechanisms and advancements to address the escalating demand for nutritious, affordable, and safe food. Despite these efforts, persistent challenges at regional levels, including climate change, degradation of water and land quality, and the preservation of biodiversity, continue to impede the fulfillment of this demand. This challenge is highlighted in a study by Manikas, Ali^5^ where the authors specify that addressing environmental issues and promoting the sustainability of food security is a multifaceted endeavor. Cernev and Fenner^6^ indicate that ensuring adequate access to nutritious food encompasses economic, environmental, and social dimensions of sustainable development. As a result, the attainment of food security is in line with several Sustainable Development Goals (SDGs). These goals encompass the eradication of poverty (SDG 1), enhancement of public health and well-being (SDG 3), and the achievement of gender equality (SDG 5), as indicated in prior research studies^7–9^.

The latest African Union's Comprehensive African Agriculture Development Programme (CAADP) biennial review report covering 2019–2021 reveals that Africa is not currently on course to achieve its goal of eradicating hunger by 2025^10^. The continent faces significant challenges in ensuring stable access to adequate, nutritious food for its growing population. According to these reports, food insecurity in Africa remains widespread and progress toward ending hunger has been insufficient. More urgent action is needed to improve food and nutrition security in Africa^10^.

To develop effective solutions for Africa's food and nutrition insecurity challenges in Africa, it is important to identify issues specific to the continent. Past research has shown a tendency to focus heavily on indicators related to food availability and accessibility^5^. However, an important observation is that few studies have incorporated utilization indicators alongside availability and accessibility indicators in their analyses. This gap is highlighted in a review conducted by Nicholson et al.^11^ examining over 90 papers on household food security models and 26 on regional models from the past decade. Additional relevant works that have evaluated food security and nutrition (FSN) in Africa include studies by Waha, Van Wijk^12^, Yuan, NourEldeen^13^, Li and Zhang^14^, Wegenast and Beck^15^, Bi, Wan^16^and Egbebiyi, Lennard^17^.

Nicholson et al.^11^ unveiled that among the four classifications of metrics (dimensions) employed to measure food security, the utilization dimension was notably underused, and only a limited number of studies applied statistical learning to identify the significant components (factors) of FSN. Additionally, the review emphasized that the majority of research in this area focused on the household level, particularly in the context of Africa. This evaluation brought to light the scarcity of studies conducted at a regional level, raising concerns about the existing gap that requires attention. Therefore, the review underscores the persisting limitations in developing comprehensive, evidence-based insights that encompass all four dimensions in the analysis of FSN in Africa.

Many traditional models^7^ do not adequately capture the spatial and temporal dynamic dependencies inherent in food security data. The current lack of dynamic models that account for changing conditions of FSN components over time and space limits the ability to develop targeted and effective food security policies. Integrating diverse datasets and analyzing them within a cohesive model poses significant challenges. By addressing these research problems, this study aims to provide valuable insights and practical solutions to enhance food security in Africa.

Dynamic spatio-temporal modeling of the interrelated factors influencing FSN over time in Africa is crucial, given the multifaceted nature of the FSN challenge, with its factors evolving both spatial and temporal. Dynamic spatio-temporal models address the limitations of traditional static modeling techniques^11,16–19^ by accounting for the spatio temporal interaction dynamic evolution of components in modeling an outcome. The research scope encompasses various dimensions, including countries^16,20^, ecosystems^21^, climate^20^, Coarse-grained reconfigurable architectures^22^, modeling infectious diseases^23^, transportation^24^ and more. However, despite numerous evaluation studies, scant attention has been given to food security, particularly in the context of Africa. To the best of our knowledge, there is a notable absence of research from a spatio-temporal dynamic perspective in this crucial area. Moreover, considering the dynamic and evolving nature of Africa's population, socioeconomics, climate, and political stability, the adoption of a dynamic spatio-temporal modeling technique becomes imperative. Hence, the application of dynamic spatio-temporal modeling within the Bayesian^25,26^ modeling framework which provides a flexible and comprehensive framework to address the uncertainties and complexities in modelling FSN concerning Africa over space and time is employed.

Unlike traditional static models, our study employs a dynamic spatio-temporal modeling approach, allowing us to capture the evolving patterns of food security over time and space. This provides a more accurate and comprehensive analysis of the underlying factors influencing food security in Africa. Our multi-dimensional approach enables a holistic analysis of the factors affecting food security, unlike many studies that focus on a single type of data or a limited set of variables. The implementation of Markov Chain Monte Carlo (MCMC) methods for parameter estimation ensures accurate and reliable model fitting. By incorporating dynamic elements and a wide range of variables, our model provides deeper insights. This holistic understanding is crucial for developing effective policies and interventions.

### Data and statistical method

The Food and Agriculture Organization (FAO) plays a central role in working towards and monitoring the achievement of Sustainable Development Goal 2 (SDG 2) which is to eliminate hunger by the year 2030. Given its integral connection with global food and agriculture, FAO serves as a primary source of data. In this study, data spanning from the year 2000 to 2019 was obtained from FAO available at http://www.fao.org/faostat/en/#home (retrieved in June 2021). Across the 20 years, our dataset comprised 54 spatial units, resulting in a total of 1080 observations. FAO uses the Food Insecurity Experience Scale (FIES) to assess the levels of food insecurity. Using adult samples within each country, FAO then calibrates the national FIES findings to a single standardized global scale. This calibration process ensures that food insecurity metrics can be comparatively evaluated at an international level^27^. To address missing values in the dataset, we employed missForest, a random forest technique, detailed in our previous work^28^. The variables used in this study have been identified as essential indicators of food security by the FAO.

We have provided explicit definitions and theoretical foundations for each variable in our earlier works, which include detailed hypotheses regarding the expected relationships between these variables and the outcomes of interest^29^. For further details, readers are referred to our previous publications^18,30^, where these variables and their theoretical frameworks are comprehensively discussed. A core indicator for examining food security and nutrition circumstances regionally is the frequency of severe food insecurity population^29^. The response metric used for this study is the number of severely food-insecure individuals. Severe food insecurity refers to scenarios where people have major risks of food shortages, hunger, and in the most problematic situations, extended durations without satisfactory access to meals^29^.

Nicholson et al.^11^, recommend that further efforts are required to build a comprehensive perspective of food insecurity and nutrition challenges in Africa. Analytical frameworks and empirical modeling should give balanced attention to indicators capturing aspects of food utilization, not just availability. While measurement is pivotal for evaluation and tracking food security status, it can be challenging to discern which dimensions, components, or levels of food security the abundant existing metrics reflect^5^. Afridi, Jabbar^31^ emphasized using a combination of evidence that brings together multiple metrics to identify the key influences on food security and nutrition. To address potential data loss and manage correlations (multicollinearity) within the original set of 40 variables (Supplementary Table 1), Principal Components Analysis (PCA) was utilized. This statistical approach aimed to avoid weaknesses linked to traditional variable selection, which usually relies on expert opinion to determine covariates. By applying PCA, the study intended a more data-driven and impartial means of identifying important covariates tied to food security and nutrition outcomes. This reduced dependence on subjective judgments. Ten factors ultimately accounted for 74.6% of the total variation in the dataset and were selected as explanatory variables. These factors covered nutrient intake, food supply, consumption status, childcare, calorie losses, environment, undernourishment, food stability, adequate diet, and infant feeding practices^18^.

The Kaiser-Meyer-Olkin (KMO) value of 0.729 and the statistically significant p-value (0.00) from Bartlett's Test of Sphericity supported using Principal Component Analysis (PCA), consistent with Bartlett^32^ recommendation. Eigenvalues greater than one were used as the standard to select principal components for extraction. To improve the clarity of each main component and ensure they provided distinct information, a Varimax orthogonal rotation method was applied, aligning with Kaiser^33^ approach.

The first principal component (PC1), accounting for 19.26% of the variance, predominantly characterizes nutrient intake. Meanwhile, PC2 explains 14.96% of the variance and represents the average food supply in Africa through factors like food supply, production, and dietary energy. PC3 reflects consumption status across Africa, incorporating indicators such as GDP per capita, childhood stunting, overweight, and adult obesity prevalence, explaining 10.96% of the variation.

PC4 accounts for 5.71% of the variability and includes factors related to child care, such as the average dietary energy requirement, the frequency of children under 5 years of age who are stunted, and the number of women of reproductive age (15–49 years) affected by anemia.

PC5 is associated with caloric loss and encompasses factors like the minimum dietary energy requirement and the incidence of caloric losses at the retail distribution level, contributing to 5.51% of the variance.PC6 represents environmental influences and accounts for 4.47% of the variance. It is associated with factors such as per capita food production variability, the percentage of the population using safely managed drinking water services, and the percentage of the population using safely managed sanitation services. PC7 represents undernourishment and contributes to 3.72% of the variance. It is linked to factors such as the prevalence of undernourishment and the number of people undernourished. PC8, associated with factors such as rail lines density (total route in km per 100 square km of land area), the percent of arable land equipped for irrigation, and the value of food imports in total merchandise exports, represents food stability and accounts for 3.43% of the variance. PC9, identified by average dietary energy supply adequacy and per capita food supply variability, signifies dietary adequacy and contributes to 3.39% of the variance. PC10, which reflects infant feeding practices with factors such as the percentage of children under 5 years affected by wasting and the prevalence of exclusive breastfeeding among infants 0–5 months of age, accounts for 3.19% of the variance.

### Spatio-temporal dynamic model

Spatial–temporal dynamic models are important when analyzing food security data that displays shifting trends or characteristics over successive periods and geographical regions. They are created to identify and adjust to alterations in the process that generate the changing values in the spatially referenced data over time^34,35^.

The formulation of the model is based on the Bayesian spatio-temporal dynamic model framework^3^. The response variable is represented as ${Y}_{{s}_{i}t}$ refers to the number of severely food insecure individuals observed across the 54 spatial units $(i)$ over time $(t = 1,...,20).$ In this context,${s}_{i}$ denotes the ${i}^{th}$ spatial location among the total of 54 units, while $t$ indexes the time period, ranging from years 1 to 20. Together, ${s}_{i}$ and $t$ specify the spatio-temporal indices for the observed responses Y that will be modeled using the Bayesian spatio-temporal dynamic framework.

1 $${Y}_{{s}_{i}t}={\mu }_{{s}_{i}t}+{\varepsilon }_{{s}_{i}t}, i=1,\dots ,54, t=1,\dots ,20$$

The model represents the response ${Y}_{{s}_{i}t}$ as comprising two components: ${\mu }_{{s}_{i}t}$, a spatiotemporal process describing the mean structure, and ${\varepsilon }_{{s}_{i}t},$ a zero-mean spatiotemporal error process. The mean structure is modeled via a regression approach as: : ${\mu }_{{s}_{i}t}={{\varvec{X}}}_{{s}_{i}t}{{\varvec{\beta}}}_{{s}_{i}t}$, where ${{\varvec{X}}}_{{s}_{i}t}$ are the available covariates and ${{\varvec{\beta}}}_{{s}_{i}t}$, are their associated coefficients. This regression component is designed to capture overall spatial, temporal, and or spatial–temporal trends present within the data.

To enable varying regression coefficients over space and time for the spatiotemporal dynamic model, Eq. (1) is expanded. Dynamic prior distributions are introduced for the regression coefficients to allow them to vary. Additionally, the spatiotemporal random effect is assigned a Gaussian process (GP) prior, but with a different correlation structure specified at each time point. The incorporation of dynamic priors and time-varying GP correlations makes the model capable of adapting to changes in both the coefficient estimates and temporal/spatial dependence patterns in the residuals. These are generated using transition equations, capturing their Markovian time dependence^3^.

The hierarchical formulation of the dynamic spatio-temporal model is expressed as follows:

2 $${Y}_{{s}_{i}t}={{\varvec{X}}}_{{s}_{i}t}{\beta }_{t}+{\aleph }_{{s}_{i}t}+{\varepsilon }_{{s}_{i}t},{ \varepsilon }_{{s}_{i}t}\sim N\left(0,{{\sigma }^{2}}_{\varepsilon t}\right) $$

3 $${\beta }_{t}={\beta }_{t-1}+{{\varvec{\eta}}}_{t}, {\boldsymbol{ }\boldsymbol{ }\boldsymbol{ }\boldsymbol{ }\boldsymbol{ }\boldsymbol{ }\boldsymbol{ }\boldsymbol{ }{\varvec{\eta}}}_{t}\sim N(0,{\sum }_{n})$$

4 $$ \aleph_{{s_{i} t}} = \aleph \left( {s_{i} ,t - 1} \right) + \omega_{{s_{i} t, }} \omega_{{s_{i} t}} \sim GP(0,z_{t} \left( { \cdot {|}\varphi_{t} )} \right) $$

The model components are specified independently for all possible combinations of the spatial index $i$, ranging from $1 to 54$, and temporal index $t$, ranging from $1 to 20.$ The model components are elucidated in the sequel.

- The original model given Eq. (1) has been expanded further to Eq. (2) with the inclusion of a time-varying variance parameter ${{\sigma }^{2}}_{\varepsilon t}$ and the spatiotemporal error term ${\varepsilon }_{{s}_{i}t}$. This allows the model to account for changes in the magnitude of random errors at different time points.
- In Eq. (3), the model has been further updated to make the regression coefficients ${\beta }_{t}$ dynamic over time. Where ${\beta }_{t-1}$ is coefficients at time *t*-*1* is based on its value at the previous time plus some Innovation term ${{\varvec{\eta}}}_{t}$ for the regression coefficients. This aids the model in capturing the inherent randomness and fluctuations in the temporal variations of the coefficients. ${\beta }_{t}$ coefficients are given a multivariate normal $N(0,{\sum }_{n})$*.* The hyper-parameter ${\sum }_{n}$ prior is also given inverse Wishart prior distribution. This helps prevent overfitting and encourages the estimation of more stable and plausible covariance structures.
- In Eq. 4, the spatio-temporal random effects ${\aleph }_{{s}_{i}t}$ are modeled using a Gaussian process. The innovation (new random effects) ${\omega }_{{s}_{i}t,}$ are assigned a time-varying Gaussian process (GP) prior. The GP has a time-varying covariance function ${z}_{t}\left(\bullet |{\varphi }_{t}\right)$ parameterized by ${\varphi }_{t}$. This allows the variance ${{\sigma }^{2}}_{\omega t}$ and correlation parameters ${\boldsymbol{\vartheta }}_{t}$ to differ at each time point $t$. These parameters enable the model to capture changes in variability and spatial correlation over time.

The model is finalized by assuming distributions for the initialization of dynamic parameters and prior distributions for the model parameters. Firstly, The initial regression parameter ${\beta }_{0}$ are presumed to follow a normal distribution ${N({\varvec{d}}}_{0}{\sum }_{0})$, where ${{\varvec{d}}}_{0}$ and ${\sum }_{0}$ are appropriately selected hyper-parameters to denote a vague prior distribution. These hyper-parameters are selected in a way that does not strongly influence the results, allowing the data to drive the posterior distribution. Secondly, The initial random effect ${\aleph }_{{s}_{i}t}$ is assumed to be **0**. This simplifies the beginning by starting the random effect ${\aleph }_{{s}_{i}t}$ at zero, providing a neutral starting point for the dynamic random effects. Thirdly, for the inverse of the variance components ${{\sigma }^{2}}_{\omega t}$ and ${{\sigma }^{2}}_{\varepsilon t}$, Gamma prior distributions $\left(\frac{1}{ {{\sigma }^{2}}_{\omega t}}\sim Gama\left({a}_{wt},{b}_{wt}\right)and \frac{1}{ {{\sigma }^{2}}_{\varepsilon t}}\sim Gama({a}_{\varepsilon t},{b}_{\varepsilon t})\right)$ are specified on their values. Gamma prior are flexible and can represent a wide range of prior beliefs. Finally, similar prior distributions are assumed for the correlation structure parameters ${\boldsymbol{\vartheta }}_{t}$, following the same principles as the priors for the variance components (${{\sigma }^{2}}_{\omega t}$ and ${{\sigma }^{2}}_{\varepsilon t}$). This maintains consistency in the prior specifications, resulting in a well-defined Bayesian hierarchical model and also producing reasonable dependence structures^3,36^.

Markov Chain Monte Carlo (MCMC) techniques are employed to construct a Markov chain that converges to the target posterior distribution. At each iteration of MCMC, a new sample is proposed from a distribution dependent on the current state of the chain. The acceptability of this proposed sample is assessed by comparing its probability to the probability of the current state. If the proposed sample has a greater probability, it is accepted. Otherwise, its acceptance is determined by the ratio of the probabilities^3^. Using R statistical software version 4.3.2, MCMC is run for 5000 iterations. The level of uncertainty in the model estimates is then quantified by computing 95% credible intervals from the posterior samples obtained via MCMC. This approach utilizes standard MCMC methodology to iteratively approximate the posterior distribution given the hierarchical Bayesian model specification and collect posterior samples for inference.

## Results

Leveraging a refined set of ten explanatory variables derived from PCA, extracted from an initial pool of 40 metrics sourced from the FAO, our study advances beyond traditional static models. Due to the spatio-temporal dynamic nature of all parameters in this model, it was not feasible to generate a comprehensive table of parameter estimates. However, our findings are concisely summarised in charts, the visual representation of dynamic coefficients, spatial–temporal dependencies, and error terms serves as a powerful tool for conveying our analytical outcomes.

### Variability measures

**Figure 1:**
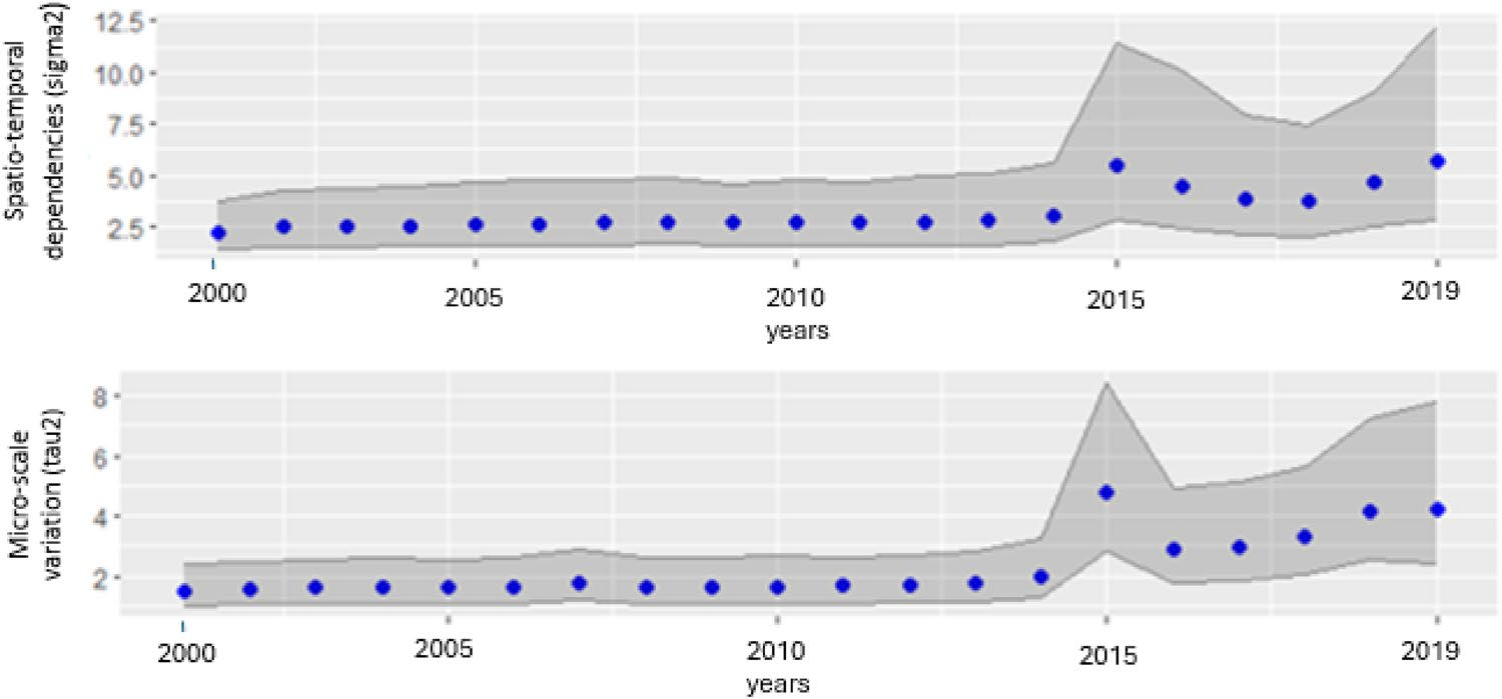
Spatio-temporal dependencies (sigma2) and micro-scale variation (tau2) for dynamic spatio-temporal modeling of food security and nutrition in Africa from 2000–2019, illustrating the overall spatiotemporal variability and unstructured spatial variability.

The upper panel of Fig. 1 shows the spatiotemporal variability in FSN across Africa over time. It represents changing patterns in the distribution of food security continent-wide. The analysis observed consistent variance from 2000 to 2014, signifying relative stability and homogeneity in FSN situations across Africa during this period. The variance peaked in 2015 but declined slightly in 2016, 2017 and 2018. However, an increasing variance from 2015 to 2019 suggests that influences on food security were becoming more dynamic and diverse over this latter period. This points to more pronounced shifts occurring in food security conditions within Africa. This rising spatiotemporal variation implies food security was experiencing changing, less uniform impacts across the region in recent years compared to the earlier period of low, stable differences seen from 2000 to 2014.

The lower panel of Fig. 1 measures the micro-scale variation (tau2), which represents the unstructured spatial variability not explained by the specified spatial structure in the dynamic spatiotemporal model. It was observed that the micro-scale variability remained fairly stable from 2000 to 2014 but increased from 2015 to 2019, peaking in 2015 in terms of FSN as illustrated in Fig. 1. This indicates that the magnitude of unexplained variability grew over the study period. This may be because country- or local-level factors and conditions evolving over time were not accounted for in the spatiotemporal model. Possible factors could include micro-scale environmental changes or community-specific dynamics not captured by the model covariates.

### Dynamic coefficients

**Figure 2:**
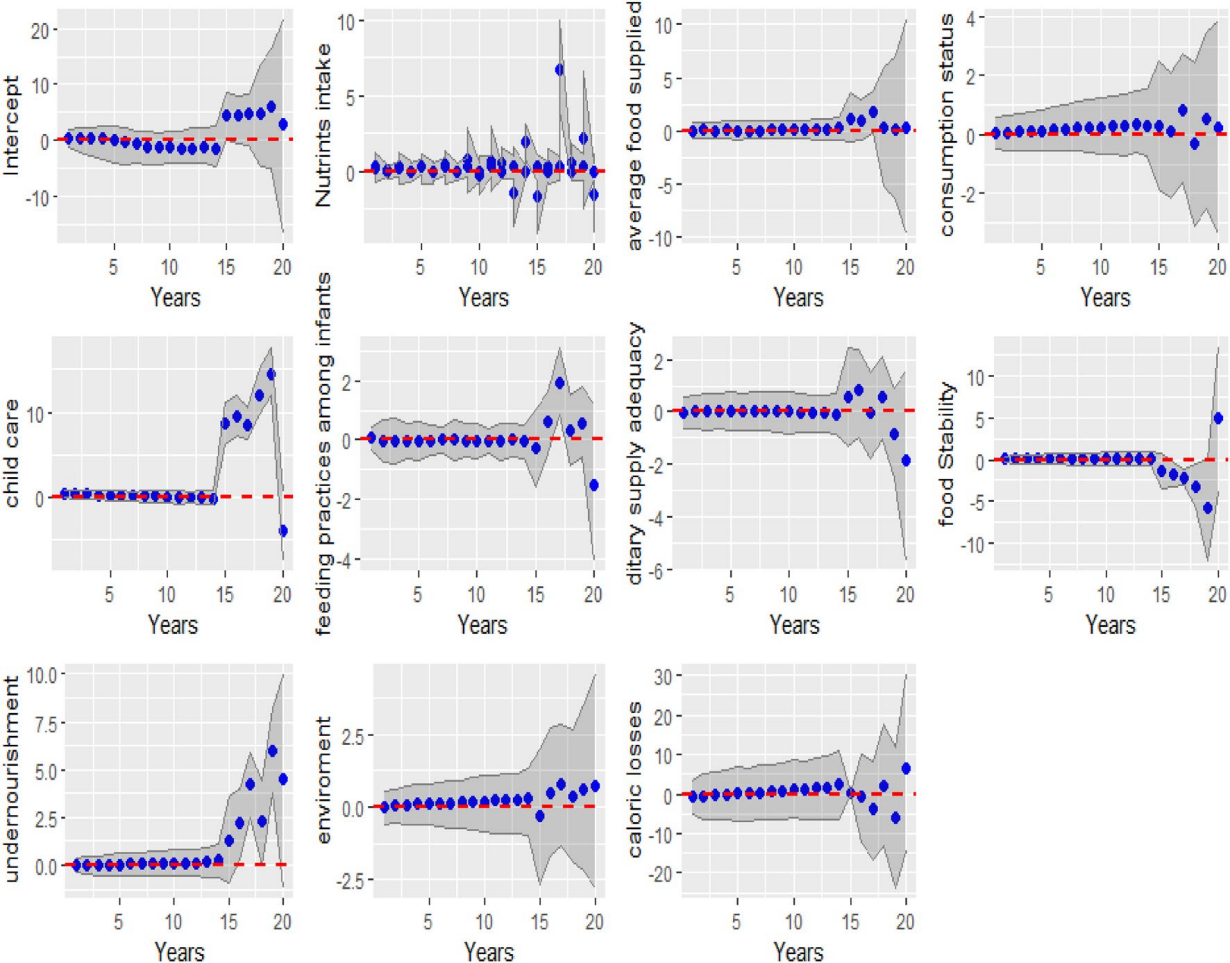
Graph depicting dynamic coefficients of food security and nutrition components in Africa from 2000 to 2019, with the Y-axis representing these coefficients and highlighting their impact on food security over time.

Figure 2 depicts the dynamic coefficients for the components of FSN in Africa. In this graphical exposition, we examine the ebb and flow of coefficients associated with the component of FSN. From the intercept, capturing the baseline conditions, to each component providing unique insights into the complex system, the graphs offer a dynamic portrayal of how these components influence food security outcomes over the two transformative decades. X-axis (Time): Reflects the years ranging from 2000 to 2019. Y-axis (Coefficient Value): Depicts the dynamic coefficients corresponding to the explanatory factors.

The coefficients associated with the component Child Care demonstrated stability from 2000 to 2014. However, between 2015 and 2018, there was a notable sharp increase, followed by a decline in 2019. This pattern suggests a reduction in the influence of child care on food security compared to the preceding years. The consistently stable dynamic coefficients from 2000 to 2014 imply a steady and moderate impact of child care on food security. Conversely, the statistically significant positive shift observed from 2015 to 2019 indicates that, during this period, child care became a more impactful factor contributing to food security in Africa. Additionally, a similar trend was observed regarding the component(factor) Undernourishment. From 2016 to 2019, there was a statistically significant impact on food security, indicating that undernourishment also became a more influential factor in contributing to food security in Africa during that period as illustrated in Fig. 2.

During the study period from 2000 to 2019, the variable nutrient intake emerges as the most volatile component among all elements influencing food security in Africa. The dynamic coefficients for nutrient intake exhibit notable fluctuations, signifying substantial variability in its impact on food security over time. The statistical analysis reveals that, unlike other components, nutrient intake experiences significant temporal variations, with periods of heightened influence followed by downturns. This heightened volatility, as evidenced by a relatively larger coefficient range and increased uncertainty, underscores the dynamic nature of the relationship between nutrient intake and food security outcomes.

As observed in Fig. 2, the analysis of dietary supply adequacy revealed noteworthy patterns. Between 2015 and 2019, the environmental factor exhibited a positive association with FSN while dietary supply adequacy showed a negative association. Additionally, there was a negative association with food stability from 2015 to 2018, turning positive in 2019. The average food supply remained relatively stable throughout the study period.

**Figure 3:**
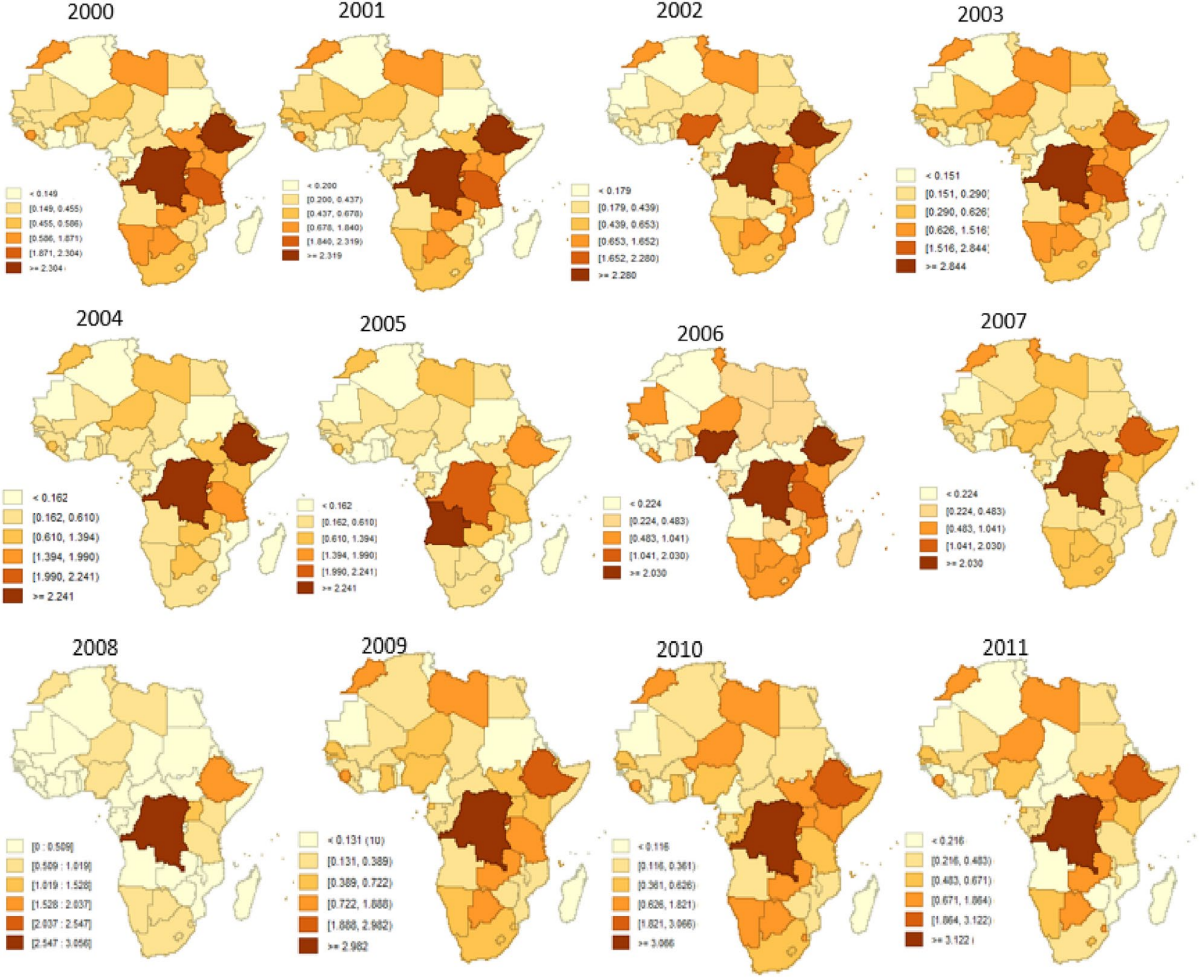

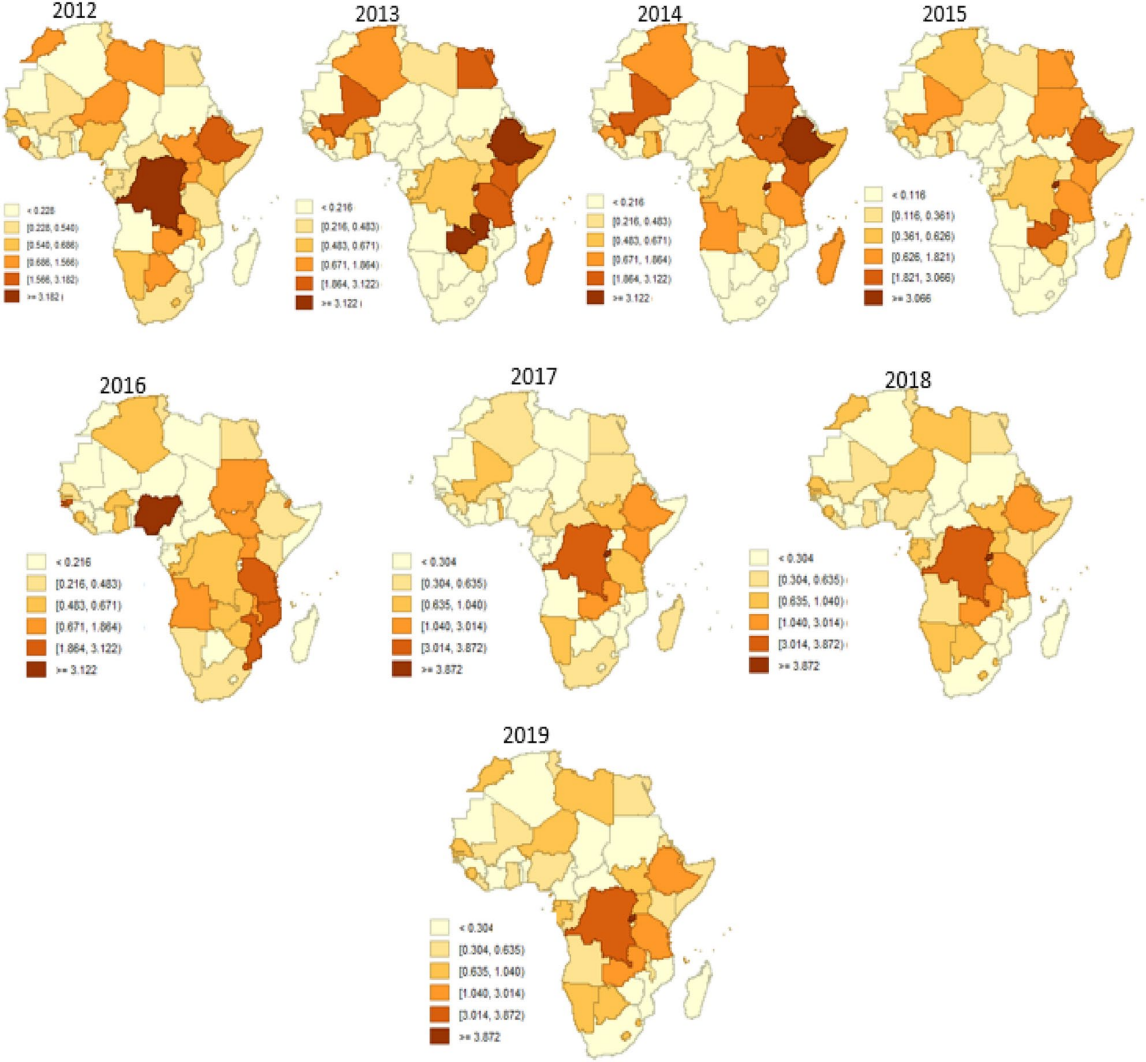
Annual maps depicting food security and nutrition trends across Africa from 2000 to 2019, highlighting regional variations and temporal changes crucial for policy and intervention strategies. Created by the corresponding author using GeoDa version 1.20.0.36 (https://geodacenter.github.io/download.html).

**Figure 4:**
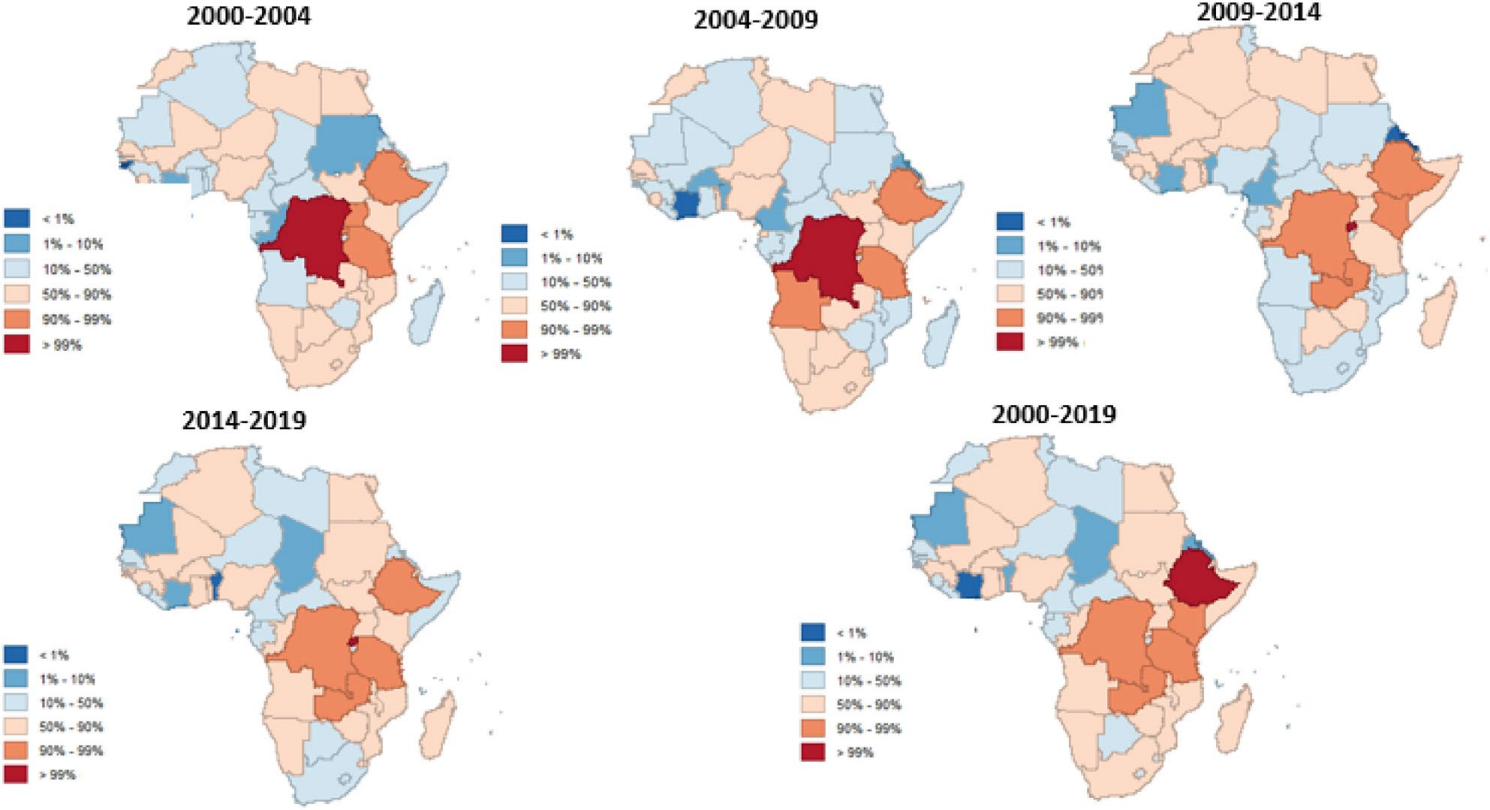
Aggregated maps illustrating food security and nutrition trends in Africa over continuous periods: 2000–2004, 2005–2009, 2010–2014, and 2015–2019. These maps offer a synthesized view of regional variations and changes over time, supporting targeted interventions and strategic resource allocation. Also Created by the corresponding author using GeoDa version 1.20.0.36 (https://geodacenter.github.io/download.html).

In Fig. 3 we employed the fitted mean values, offering a straightforward depiction of the average conditions across Africa. However, to capture the spatial dynamism, a percentile plot is utilized for Fig. 4. This approach is justified by its ability to effectively showcase aggregated patterns over time, transcending singular mean values and providing a more comprehensive view of the variability and trends within Africa. Notably, the use of a percentiles plot here is advantageous in that it is less affected by outliers^37,38^, which may arise due to the aggregation of means. This ensures a more robust representation of the spatial dynamism of FSN, offering a visual tool that is resistant to the influence of extreme values. Figures 3 and 4 are clearly labeled and include legends to aid interpretation, where deeper colors indicate a higher incidence of FSN. We created the maps in Figs. 3 and 4 using GeoDa version 1.20.0.36, a free open-source software tool https://geodacenter.github.io/download.html^39^.

### Continuous time series FSN maps of Africa

The analysis of continuous time series maps of Fig. 3 enabled an examination of the yearly changes in each FSN level across Africa. This approach facilitated the identification of trends and characteristics associated with the evolving dynamics of FSN over time. The spatial distribution of Africa's FSN over a continuous 20-year period, spanning from 2000 to 2019, is illustrated in Fig. 3. The continuous time series reveals a relatively stable variation throughout the study period, with minimal changes from 2000 to 2003. In the subsequent period, from 2004 to 2008, slight alterations in the spatial distribution are observed. Additionally, from 2009 to 2014, notable changes in FSN are evident, particularly in the Northern, Central, and Eastern parts of Africa. The distribution remains relatively consistent with minimal changes in the last three years of the study period, with notable shifts in 2015 and 2016.

### Spatial dynamisity of FSN for the various periods (2000–2004,2004–2009,2009–2014,2014–2019)

Figure 4 shows the analysis of FSN dynamicity across African countries revealing distinct chronological progressions of spatial patterns over the study period (2000–2019). Generally, high levels of FSN dynamicity are prevalent in the eastern, southern, and central regions, while isolated instances of moderate to low levels are observed in the west and north. The FSN dynamic pattern varies at different stages, with notable concentrations of high dynamicity observed in countries such as the Democratic Republic of Congo, Zambia, Tanzania, Kenya, Ethiopia, Angola, Namibia, Niger, and Nigeria across multiple stages (2000–2004, 2009–2014, and 2014–2019). Particularly, the Democratic Republic of Congo and Ethiopia consistently exhibit high levels of FSN dynamicity throughout the study period. Nigeria, on the other hand, shows varying but relatively high dynamicity at certain stages (2000–2004, 2004–2009, 2014–2019). In contrast, countries like Ghana, Togo, Burkina Faso, Guinea, and Sierra Leone manifest high dynamicity in the middle-to-late and late stages (2009–2014 and 2014–2019), while South Africa and Senegal demonstrate high FSN dynamicity in the early and middle-to-late stages (2000–2004 and 2009–2014). This leads us to hypothesize that the dynamicity of high levels of FSN is more conspicuous, highlighting the need for targeted interventions and policy considerations across regions and stages. Policy measures and interventions could involve investing in infrastructure development, especially in transportation and storage facilities. This is crucial to streamline the effective distribution of food resources from areas with a surplus to those facing deficits in Africa.

### Model validation

**Figure 5:**
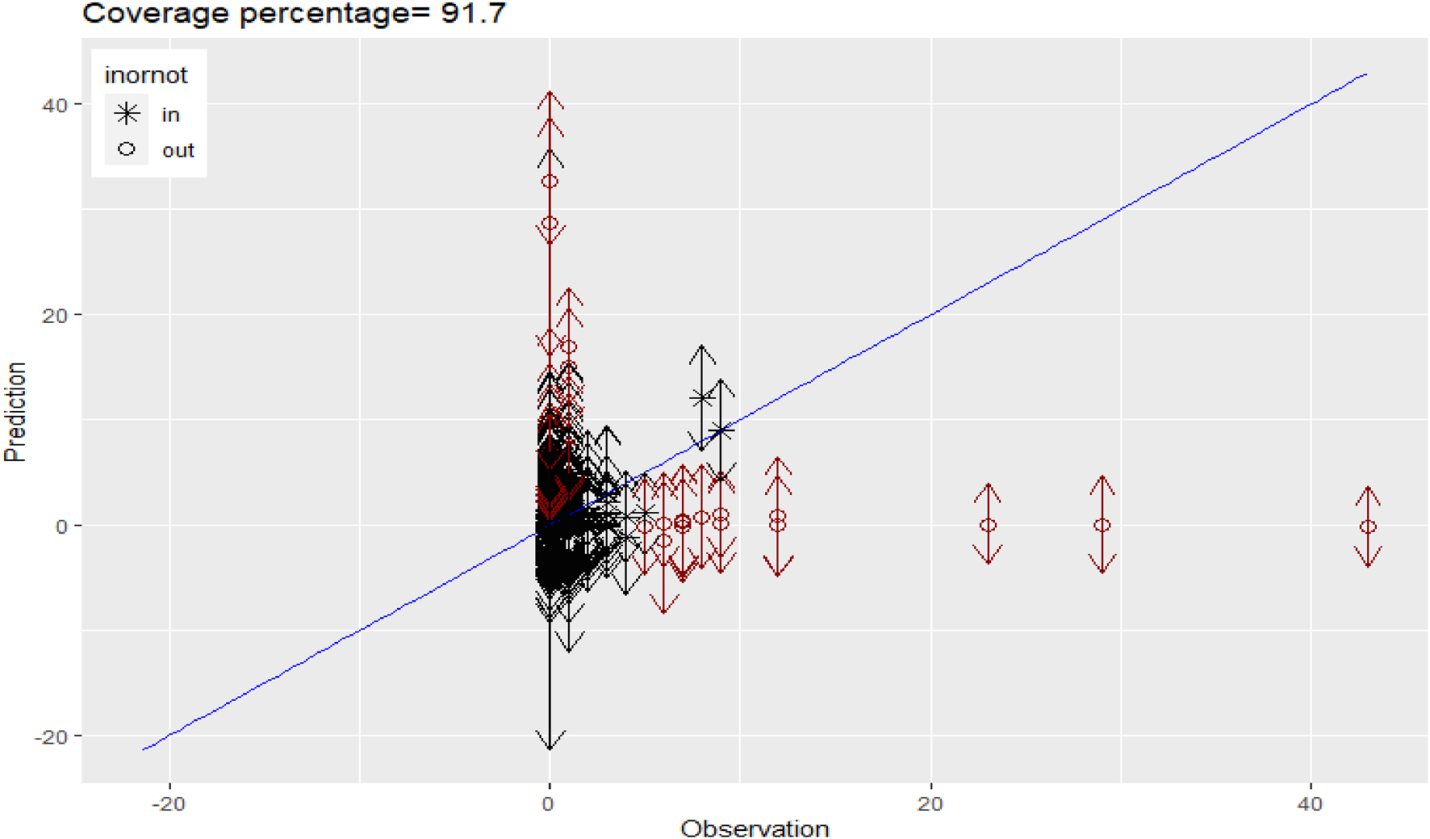
Model validation plot showing coverage percentage using a 70% training and 30% testing split. This evaluation metric illustrates the model's performance in capturing uncertainty, crucial for robust predictions and decision-making.

To ensure the robustness and generalizability of our spatio-temporal dynamic model, we performed out-of-sample testing. Specifically, the data was divided into training (70%) and testing sets (30%), ensuring that the testing set was not used during the model training phase. This methodology provides a more accurate assessment of the model's predictive capabilities on unseen data. The coverage percentage(CP) for the 95% predictive intervals was computed using the formula: $$CP=100\frac{1}{k}{\sum }_{i=1}^{k}I\left({L}_{i}\le {y}_{i}\le {U}_{i}\right)$$, , where $${y}_{i}$$ represents the observed value for $$i=1,...,k$$ and $${(L}_{i},$$ $${U}_{i})$$ represents the 100(1 − α)% predictive interval for predicting $${y}_{i}$$. The indicator function I(·) was also utilized in the coverage percentage calculation. The coverage percentage metric, as shown in Fig. 5, achieved a value of 91.7%. This high coverage percentage indicates that our model performs well in predicting food security and nutrition in Africa within an acceptable range, thereby demonstrating its reliability and robustness. It supports the significance of considering the dynamic effect in explaining food security and nutrition across the continent.

## Discussion

Utilizing dynamic spatio-temporal modeling allows for the consideration of spatial variability. It captures the temporal dynamics, enabling an examination of how the components of FSN evolve over time to facilitate the identification of trends and turning points. By integrating various interconnected factors that influence FSN, the model offers a comprehensive understanding of the intricate dynamics involved. Moreover, it assists in pinpointing geographical hotspots, contributing valuable insights for policy assessments and proactive planning to address future trends in FSN. In this work, the dynamic modeling techniques we employed followed the procedure outlined by Sahu^3^ Our choice of the current model was carefully guided by recent literature highlighting its appropriateness and effectiveness in similar contexts. This model has been recognized for its capability to accommodate temporal dependencies and capture evolving spatial patterns, thereby offering more robust and insightful analyses compared to static models, especially dealing with FSN across Africa^3,36^.

We employed a spatio-temporal dynamic^3,40^ approach to investigate the interlinkages between the spatio-temporal dynamics of food security outcomes, specifically the number of severely food insecure individuals, and shifts in the components(factors) of FSN in the context of Africa. Through this approach, we aimed to uncover how changes in these components contribute to the comprehensive dynamics of FSN across Africa. These components were derived using Principal Component Analysis (PCA) from FAO’s food security and nutrition data^18^.

Internal variations among the 54 countries in Africa are evident in Figs. 3 and 4. Overall, the Democratic Republic of Congo, Zambia, Tanzania, Kenya, Ethiopia, Angola, Namibia, Niger, and Nigeria exhibited higher levels of active FSN compared to others. Particularly noteworthy are the Democratic Republic of Congo and Ethiopia, which consistently maintained high levels over the various periods concerning FSN dynamicity. In the case of the Central African Republic and Gabon, the built-up of FSN dynamicity was not prominently evident. Conversely, the dynamicity of high levels of FSN was more apparent across Africa, particularly displaying relatively high levels of clustering in the Eastern, Southern, and North-Eastern parts of the continent. The results highlight a consistent pattern of heightened Food Security and Nutrition (FSN) levels, displaying noteworthy stability in the initial stages, continuing through the middle-to-late period, and experiencing a pronounced acceleration in the late stage of the study period.

The intercept value, indicative of the overall average level of food security, demonstrated relative stability from 2000 to 2005. Subsequently, there was a slight decline from 2006 to 2014. However, a noteworthy increase in average food insecurity among individuals occurred from 2015 to 2019, suggesting that the baseline or typical level of food security across Africa was comparatively higher during this later period than in earlier years. The dynamic intercept trend in Fig. 2 and the spatio-temporal spatial map (Fig. 3) of FSN provide valuable regional insights into the dynamism of FSN. These insights shed light on specific periods where remedial actions taken for FSN in Africa may not have yielded the desired effectiveness^10^. This information is crucial for supporting authorities responsible for food security, as it helps them identify areas and timeframes where policy interventions aimed at reducing food security incidents across the continent may need to be refined and strengthened.

Upon comparing the dynamic coefficients, it becomes evident that both child care factors and undernourishment factors have gained relatively more influence compared to other components (factors) of FSN in recent years. Hence, it is essential to identify the particular metric(s) within the components of “Child Care” and Undernourishment, considering the Pearson correlation coefficients^41^ associated with the original metrics within the FAO data. This information is crucial for optimizing Africa's scarce resources. Specifically, the factor child care was strongly correlated(0.8) with the average dietary energy Requirement^42^, the frequency of Children under 5 years of age who are stunted^42,43^, and the number of women of reproductive age (15–49 years) affected by anemia. Similarly, undernourishment had a strong correlation (0.8) with the prevalence of undernourishment and the number of people undernourished.

The findings from this study offer valuable insights into the dynamics of food security, informing future policies. This knowledge will guide more targeted interventions, improving FSN, and optimizing funding for interventions. Firstly, our spatio-temporal dynamics uncovered countries within the region facing persistent challenges or undergoing rapid changes. This enables policymakers to allocate resources to specific areas for optimal resource utilization. Additionally, there is a need to encourage collaboration between different sectors (health, education, agriculture, and social services) to develop integrated policies responsive to changing conditions in Africa. Furthermore, our findings emphasize the essential need to implement real-time monitoring mechanisms to promptly detect shifts in FSN indicators concerning Africa. Policymakers must formulate strategies addressing immediate challenges and anticipating future trends, fostering sustainable improvements in food security over time.

Although our research offers valuable insights into food security dynamics in Africa, it's important to acknowledge that the study period and geographic scope might not fully encapsulate all pertinent variations in food security dynamics. Moreover, the model outputs are susceptible to stochastic variation. Our Bayesian analysis depends on defining prior distributions, which can introduce subjectivity into the modeling process. The selection of priors could impact the posterior inference, particularly if the priors are too vague or too informative. Moving forward, it's imperative to address these limitations by enhancing data collection methods, refining models, and conducting sensitivity analyses. As with any statistical approach, careful consideration of these limitations is essential for the appropriate application and interpretation.

Depending on FAO data, despite its general reliability, presents limitations due to challenges with data availability and reproducibility in low-resource settings, as well as potential reporting biases. To mitigate these issues, it's essential to consider the food security variables as outlined in "Global Map and Indicators of Food System Sustainability"^44^, and explore alternative data sources and methodologies, such as those examined in "Multi-Dimensional Dataset of Open Data and Satellite Images for Characterization of Food Security and Nutrition"^45^, or "Rising tensions along the agri-food value chains during the COVID-19 crisis: evidence based on Google Trends Data"^46^. Future research could leverage these alternative data sources to gain insights into the diverse data requirements and explore alternative approaches across different contexts.

## Conclusion

In our study, we have illuminated the influence of factors shaping food security in Africa, specifically spanning the years 2000 to 2019. Utilizing regularly collected national FAO data and employing principal component analysis, we derived the most informative components, enabling a regional spatio-temporal dynamic modeling of food security and nutrition in Africa. While many studies employ spatial models to identify significant covariates of food security and nutrition, our approach acknowledges and reveals the dynamic nature of food security. Notably, our findings underscore that the most significant components affecting the dynamic of food security in Africa are child care and undernourishment. This insight is pertinent for policymakers dealing with limited resources, offering valuable guidance in formulating effective policies to address food security challenges across the continent.

### Ethics approval and consent to participate

The Suite of Food Security Indicators presents a fundamental collection of indicators focused on food security. This selection is based on the recommendations of experts who convened during the Committee on World Food Security (CFS) Round Table on hunger measurement at FAO headquarters in September 2011. These indicators were chosen to encompass various dimensions of food insecurity. The selection process considered expert insights and the availability of data that permits cross-regional and temporal comparisons. The FAO Statistical Quality Assurance Framework's Principle 10 underscores the strict confidentiality of data subject to national policies, particularly regarding individuals, entities, and small aggregates. Such data are exclusively used for statistical purposes or in accordance with legal mandates.

### Supplementary Information

Supplementary Table 1.

## Data Availability

The datasets used and/or analysed during the current study available from the corresponding author on reasonable request.
