# Supplementary material for: A bayesian spatio-temporal dynamic analysis of food security in Africa
Source: Sci Rep. 2024 Jul 2;14:15132. doi: 10.1038/s41598-024-65989-z (PMC11219794; doi:10.1038/s41598-024-65989-z)
Supplement: Supplementary file 1 — Supplementary Table 1. [file 41598_2024_65989_MOESM1_ESM.docx]

**Supplementary Table 1: Food security and nutrition indicators from FAO**

| **Variable Name** | **Definition of variables** |
| --- | --- |
| v1 | Average dietary energy supply adequacy |
| v2 | Average value of food production (constant 2004-2006 I$/cap) |
| v3 | Dietary energy supply used in the estimation of prevalence of undernourishment |
| v4 | Share of dietary energy supply derived from cereals, roots and tubers (kcal/cap/day) |
| v5 | Average protein supply (g/cap/day) |
| v6 | Average supply of protein of animal origin (g/cap/day) |
| v7 | Rail lines density (total route in km per 100 square km of land area) |
| v8 | Gross domestic product per capita, PPP, dissemination (constant 2011 international $) |
| v9 | Prevalence of undernourishment (percent) |
| v10 | Number of people undernourished (million) |
| v11 | Prevalence of severe food insecurity in the total population (percent) |
| v12 | Prevalence of moderate or severe food insecurity in the total population |
| v13 | Cereal import dependency ratio (percent) |
| v14 | Percent of arable land equipped for irrigation (percent) (3-year average) |
| v15 | Value of food imports in total merchandise exports |
| v16 | Political stability and absence of violence/terrorism (index) |
| v17 | Per capita food production variability (constant 2004-2006 thousand int$ per capita) |
| v18 | Per capita food supply variability (kcal/cap/day) |
| v19 | Percentage of population using safely managed drinking water services |
| v20 | Percentage of population using at least basic drinking water services |
| v21 | Percentage of population using safely managed sanitation services |
| v22 | Percentage of population using at least basic sanitation services |
| v23 | Percentage of children under 5 years affected by wasting |
| v24 | Number of children under 5 years affected by wasting (million) |
| v25 | Percentage of children under 5 years of age who are stunted (modelled estimates) |
| v26 | Number of children under 5 years of age who are stunted (modeled estimates) |
| v27 | Percentage of children under 5 years of age who are overweight (modeled estimates) |
| v28 | Number of children under 5 years of age who are overweight (modeled estimates) |
| v29 | Prevalence of obesity in the adult population (18 years and older) |
| v30 | Number of obese adults (18 years and older) (million) |
| v31 | Prevalence of anemia among women of reproductive age (15-49 years) |
| v32 | Number of women of reproductive age (15-49 years) affected by anemia (million) |
| v33 | Prevalence of exclusive breastfeeding among infants 0-5 months of age |
| v34 | Prevalence of low birth weight |
| v35 | Number of newborns with low birthweight (million) |
| v36 | Minimum dietary energy requirement (kcal/cap/day) |
| v37 | Average dietary energy requirement (kcal/cap/day) |
| v38 | Coefficient of variation of habitual caloric consumption distribution (real number) |
| v39 | Incidence of caloric losses at the retail distribution level |
| v40 | Average fat supply (g/cap/day) (3-year average) |
